# Comparison of CBCT examined root thickness and fracture resistance

**DOI:** 10.6026/9732063002001012

**Published:** 2024-09-30

**Authors:** Garima Kumari, Sibani Sarangi, Bandana Panda, Vanka Aruna, Jyoti Kasana, Priya Sinha, Ritik Kashwani

**Affiliations:** 1 Department of Conservative Dentistry and Endodontics, Manav Rachna Dental College, MRIIRS, Faridabad, India; 2 Department of Periodontology, Hitech Dental College and Hospital, Bhubaneswar, India; 3 Department of Conservative Dentistry and Endodontics, Kalinga Institute of Dental sciences, Bhubaneswar, India; 4 Department of Periodontology, Bangalore Institute of Dental Sciences, Karnataka, India; 5 North DMC Medical College and Hindu Rao Hospital, Delhi, India; 6 Buddha Institute of Dental Sciences and Hospital, Magadh University, Patna, India; 7 Department of Oral Medicine and Maxillofacial Radiology, School of Dental Sciences, Sharda University, Greater Noida

**Keywords:** CBCT, fracture resistance, Ni-Ti files, residual root thickness, universal testing machine

## Abstract

The correlation between remaining dentin thickness and fracture resistance in prepared teeth is of interest to dentists. A sample of 60 human teeth (mandibular premolars) was extracted and examined using cone beam computed tomography to assess residual dentin thickness before and after instrumentation. The gathered samples have been divided into three distinct categories, each with 20 samples. Hand files were used in Group 1, Protaper Next was used in Group 2, and a V taper was used in Group 3. They were examined for remaining dentin thickness after preparation with 3D CS Software and fracture resistance utilizing a Universal Testing Machine (UTM). Therefore, the objective of this research is to compare the amount of dentin removed by V Taper and Pro Taper Next to hand files using CBCT at the apical and coronal middle levels. Following that, these values will be correlated with fracture resistance values obtained from the Universal Testing Machine.

## Background:

Mechanical preparation of root canals using Ni-Ti rotary instruments causes vertical root fracture (VRF), which is a major problem associated with contemporary endodontic practice. Clinically, 10.9% to 31% of cases necessitate extraction either before or following endodontic therapy because of vertical root fracture [1, 2]. Among the multiple predisposing factors for VRF, the diameter of the prepared canal and the excessive taper of the rotary instruments were frequently noted as possible explanations for excessive dentin removal and root weakness; nevertheless, these remain debatable. All rotary systems used in dentistry have variable blade design, tip diameter, and configuration; however there is an important link between the Ni-Ti file system and dentinal micro fractures that lead to vertical root fracture [3, 4]. Bier *et al.* observed that root canal preparation with nickel titanium devices produced higher dentinal damage than by hand files [5]. Protaper Next with its off-set blade combined with the increasing and decreasing taper has shown to prevent taper lock thus resulting in fewer dentinal micro cracks. V Taper system with its variable taper design creates conservative coronal shape thus resulting in less dentinal removal and probably fewer dentinal micro cracks [6]. A handful of studies have been done on the dentin removal and fracture resistance of Pro Taper Next rotary instruments; however, no study has been reported to assess the fracture resistance of V taper rotary instruments. Numerous investigations verified the correlation between dentinal micro-cracks and vertical root fractures as a result of using various Ni-Ti files for instrumentation [7, 8]. Therefore, it is of interest to report the comparison of CBCT examined root thickness and fracture resistance.

## Material and Methods:

This study was approved by the Institutional Research Ethical Committee at Manav Rachna Dental College (Approval ID ACAD/2017/492) and was conducted in the Department of Conservative Dentistry and Endodontics, Manav Rachna Dental College, Faridabad. It was approved by the internal ethical committee. We acquired orthodontically extracted human mandibular premolar teeth from patients ranging in age from 17 to 24. According to the rules and protocols set forth by the Occupational Safety and Health Administration, the extracted teeth were collected, stored, sterilized, and handled. To rule out the possibility of multiple canals, radiographs were taken in the Buccolingual and Mesiodistal angulations. The teeth were inspected for any cracks or anomalies that would need their exclusion using the Dental Operating Microscope (Global A-series TM Microscope) at 10X magnification. After that, the teeth were washed and preserved for a week in 0.5% sodium hypochlorite solution. Following this, the teeth were decoronated to provide 13mm uniform root lengths.

## Sample size calculation and distribution:

With the study's power established at 80.0% [(type II error = 0.20]] and 5% Type 1 error probability [α=0.05)], a sample size of 60 was selected. Following that, using the online randomization tool www.randomiser.org, the chosen teeth were numbered and categorized into three experimental groups of 20 each.

## Mounting of teeth:

10 silicon molds, each containing six teeth (Affinis Putty, Coltene Whale dent), were utilized for positioning the teeth. Lead films were set on the right side of the mold in order to aid with the alignment of the teeth in the CBCT sections. A third person who was not participating in the study aligned the samples and allocated dental codes.

## CBCT imaging:

Each mould was subjected to the CBCT scanner (Giano. Newtom Italy), using the image protocol for teeth, with the following exposure parameter: 10 Kv, 8mA, and 9 seconds. Each section was evaluated at 4 points (mesial, distal, buccal and lingual) in CS3D CBCT software.

## Preparation of teeth:

Under the supervision of a senior faculty member with over twelve years of postgraduate teaching experience, a single operator performed the biomechanical preparation of the teeth. Six teeth were instrumented simultaneously to eliminate operator bias and fatigue. A No. 2 round carbide bur was utilized to prepare or modify the endodontic access cavity, and an Endo Access bur (DENTSPLY Endo Access Bur FG 1) was utilized to refine it. A DG 16 explorer was employed to locate the canals (Hu Friedy, IL USA). A # 10 K file was pushed 1 mm past the apical foramen and then removed to assess the canal's patency and calculate its working length. One millimetre less than the anatomic apex were the final working length.

## Group 1(n=20): Step-back technique using stainless steel K-files:

Using a quarter turn pull, K-files (Mani Inc., Japan) prepared the canals up to #40 as the master apical file (MAF), after which they stepped back in increments of 1 mm for the subsequent three sizes of files (#45, #50, and #55). Each step back size file was accompanied by recapitulation utilizing the MAF at the working length.

## Group 2: Pro Taper Next rotary files:

Using a torque-controlled endo motor (E connect S, Oricam), canals were prepared using the Protaper Next system in accordance with manufacturer guidelines up to X4 (40.06) at 300 rpm and 2 Ncm torque.

## Group 3: V taper rotary files (SS White):

Making use of the torque-controlled endo motor (E connect S, Oricam), canals were prepared up to size (40.06). After every filling, the canals were flushed with 5 milliliters of 17% EDTA for duration of one minute, after being irrigated with 2 milliliters of normal saline and 1 milliliter of 2.5% NaOCL.

## Evaluation of remaining dentin thickness:

Following canal preparation, the samples were placed back into the original mold, and CBCT scans were taken using a methodology akin to that of the original imaging. The minimal residual thickness (MRRT) was subsequently assessed. The residual root thickness (RRT) was subtracted from the initial root thickness (IRT) to determine the amount of dentin removal (DR). With the aid of the CS 3D imaging program, the data was examined.

## Evaluation of fracture resistance:

Precision balances and vernier calipers have been employed to measure the weight and Buccolingual (BL) and Mesiodistal (MD) dimensions of the roots, respectively, and to fix the measurement errors. For block preparation, acrylic resin was utilized. Each root's coronal portion was exposed about 9 mm because of the apical root end being placed 4 mm vertically in the acrylic resin. The root was fractured via universal testing equipment having a cross head speed of 1 mm/min. A conical tip made from steel with a tip diameter of 0.5 mm was fixed to each specimen and placed parallel to its long axis with the canal orifice in center. The load necessary for fracture was recorded and expressed in Newton (N).

## Results:

## Statistical analysis:

One-way ANOVAs were primarily utilized for statistical analysis, and for multiple comparisons, the Post-hoc test was implemented after ward. The data provided was shown as mean +SD. The change in relative value with regard to time was assessed using a paired t-test. P-values below 0.05 are, at a 95% confidence level, considered to be significant. The analysis was performed employing SPSS 18.0, statistical software.

## Minimum residual root thickness (MRRT):

**Figure 1:**
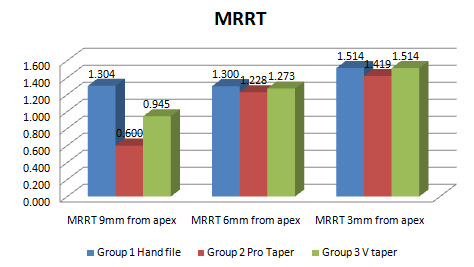
Comparison graph of MRRT

When MRRT was compared within the group, the coronal third showed a statistically significant difference whereas the middle and apical third revealed no significant change. (P<0.001) Groups 1 and 2 demonstrated a significant difference in MRRT intergroup comparison, whereas groups 1 and 3 indicated no discernible distinction. (P < 0.001) The comparative analysis of minimal residual root thickness among all the groups has been demonstrated in (Figure 1) (Table 1).

## Dentin removal (DR):

There was no statistically significant distinction between groups 1 and 3, nonetheless there was a substantial variance between the two when it related to dentin removal (DR) in the intergroup comparison (P<0.001). With group 2, dentin removal seemed more conspicuous than with group 1, which was followed by group 3. (Table 2)

Group 2 >Group 3 > Group 1

## Fracture resistance:

**Figure 2:**
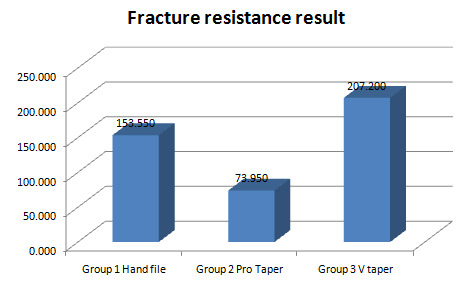
Graph of FR result

During assessment, each root examined in this study demonstrated a vertical fracture extending in the labiolingual direction. The three groups varied substantially, based on the results. Group 2 possessed the smallest degree of fracture resistance, followed by Group 1 and Group 3 showed the greatest amount of fracture resistance. (Table 3) (Figure 2)

Group 3 >Group 1 > group 2

## Discussion:

One of the most critical iatrogenic components that affect a tooth's capability to endure a fracture is the thickness of the residual dentin following root canal treatment. Recent advances in non-surgical endodontic treatment techniques have begun to support rotary instrumentation considerably. Research indicates that, in comparison to hand files made of stainless steel, rotary instruments made out of Ni-Ti alloy have improved biomechanical preparation, enhancing the efficacy of root canal preparation [9- 10]. Nevertheless, these instruments can either entirely or partially remove dentin from teeth while cleaning and shaping, weakening the tooth structure that remains and increasing the likelihood of breakage. While utilizing a highly tapered instrument for dentin removal, Zandbiglari *et al.* determined that the teeth appeared more susceptible to fracture compared to if one used hand instruments during preparation [3]. According to Lertchirakkarn *et al.* (2003), vertical root fracture wasn't caused solely by forces applied while lateral compaction [12, 13]. Thus, without obturating the prepared teeth, the study's main emphasis was on evaluating the dentin thickness which remained and the vertical root fracture. One of the newest technological advancements in radiology that can be used for research in the dentistry and medical fields is CBCT. With the capability to determine the remaining dentin thickness before as well as following instrumentation, this device provides an advantageous and non-invasive technique which holds great promise for applications in endodontic research. Thus, the primary objective of the present research investigation sought to assess the residual root thickness following instrumentation using hand and different rotary files using CBCT analysis, and investigate whether or not this thickness has an association with the tooth's capability to endure fracture. Both fracture resistance [14, 15, 16, 17, 18, 19, 20-21] and residual dentin thickness [22, 23, 24, 25, 26-27] have been evaluated individually in quite a few of studies that have been published. Only a few individuals looked at the two metrics' links [28]. Therefore, without obturating the prepared teeth, the study's main objective was on evaluating the dentin thickness which remained and vertical root fracture. Additionally, only a limited number of studies [28] have provided an in-depth assessment of dentin removal at the apical, middle, and coronal aspects-aspects which the present research has also assessed.

Protaper Next (PTN) and V Taper rotary instruments were used to prepare the canals as opposed to the hand-instrumented group. There are researches [22, 23, 24] that analyze PTN, but to as far as we have knowledge, no published research reviews the fracture resistance of V Taper rotary files. The company's representatives claim that the V Taper files' unique design is expected to result in less dentin loss, strengthening the teeth's resistance to breakage. The dentin removal for Group PTN was significantly distinct from V Taper and traditional files (P<0.001), as determined by the data. Group 1 and Group 3 were not substantially different from each other, indicating that the amount of dentin removed with a V-taper was roughly comparable to that obtained with hand files (Table 2). This is consistent with the studies conducted by Akhlagi *et al.* (2010) [6]. The more prominent taper could have contributed to the more dentin loss observed in the coronal section of the canals, judging by the data. The fracture resistance of the three groups differed substantially, based on the results (Table 3). The groups having the lowest fracture resistance were Group 2 (Pro Taper Next), Group 1 (Hand file), and Group 3 (V Taper), which showed the highest fracture resistance. This suggested that there is a significant relationship between vertical root fracture and residual dentin thickness. The null hypothesis was thus disproved. To further corroborate these findings, bigger numbers of samples for comparable research are encouraged. One of the drawbacks of this research was that, considering it was carried out *in vitro*, care had to be taken when evaluating the results for clinical use.

## Conclusion:

The study found a positive correlation between remaining dentin thickness and fracture resistance of teeth. CBCT examination revealed that V Taper rotary file systems preserved residual root thickness more effectively than hand instrumentation, with V Taper showing the highest fracture resistance. The findings suggest that rotary systems with conservative designs, such as V Taper, better preserve tooth structure and enhance fracture resistance during endodontic procedures.

## Figures and Tables

**Table 1:** The mean of the MRRT (Minimum residual root thickness) at different sections of single root mandibular premolars in all three groups (in mm)

		**N**	**MEAN**	**STD DEVIATION**	**MINIMUM**	**MAXIMUM**	**F-VALUE**	**P-VALUE**
MRRT 9mm from apex	GROUP 1 Hand File	20	1.304	0.953	0.82	5.3		
	GROUP 2 Protaper	20	0.6	0.158	0.35	0.98	7.749	0.001
	GROUP 3 VTaper	20	0.945	0.168	0.68	1.2		
	TOTAL	60	0.95	0.627	0.35	5.3		
MRRT 6mm from apex	GROUP 1 Hand File	20	1.3	0.147	1	1.58		
	GROUP 2 Protaper	20	1.228	0.581	0.65	3.5	0.205	0.815
	GROUP 3 VTaper	20	1.273	0.169	1.02	1.78		
	TOTAL	60	1.267	0.354	0.65	3.5		
MRRT 3mm from apex	GROUP 1 Hand File	20	1.514	0.167	1.2	1.8		
	GROUP 2 Protaper	20	1.419	0.18	1	1.65	2.536	0.088
	GROUP 3 VTaper	20	1.514	0.105	1.35	1.72		
	TOTAL	60	1.482	0.158	1	1.8		

**Table 2:** The mean of the DR (dentin removal) at different sections of single root mandibular premolars in all three groups (in mm)

		**N**	**Mean**	**Std. Deviation**	**Minimum**	**Maximum**	**F-value**	**p-value**
DR 9mm from apex	Group 1 Hand file	20	0.166	0.089	0.08	0.32	71.603	<0.001
	Group 2 Pro Taper	20	0.668	0.197	0.32	0.98		
	Group 3 V taper	20	0.246	0.118	0.1	0.65		
	Total	60	0.36	0.262	0.08	0.98		
DR 6mm from apex	Group 1 Hand file	20	0.199	0.114	0.08	0.42	18.43	<0.001
	Group 2 Pro Taper	20	0.428	0.2	0.2	0.85		
	Group 3 V taper	20	0.196	0.067	0.1	0.35		
	Total	60	0.274	0.174	0.08	0.85		
DR 3mm from apex	Group 1 Hand file	19	0.143	0.082	0.08	0.38	3.66	0.032
	Group 2 Pro Taper	20	0.234	0.141	0.1	0.5		
	Group 3 V taper	20	0.175	0.084	0.08	0.35		
	Total	59	0.185	0.111	0.08	0.5		

**Table 3:** Cross sectional diameters, multiplication of the BL-MD Diameters, weights, and facture loads of the roots

**Groups**	**N**	**BL**	**MD**	**Multiplication of BL and MB**	**Weight (g)**	**Fracture load (N)**
Hand file	20	7.24+0.44	5.16+0.48	37.38+3.92	0.45+0.05	153.55+0.02
Pro Taper file	20	7.16+0.39	5.18+0.36	37.12+3.53	0.43+0.03	73.95+0.6
V Taper	20	7.26+0.42	5.15+0.38	37.37+3.65	0.44+0.05	207.20+0.7
